# Unsupervised title and abstract screening for systematic review: a retrospective case-study using topic modelling methodology

**DOI:** 10.1186/s13643-022-02163-4

**Published:** 2023-01-03

**Authors:** Agnes Natukunda, Leacky K. Muchene

**Affiliations:** 1 Immunomodulation and Vaccines Programme, MRC/UVRI and LSHTM Uganda Research Unit, Entebbe, Uganda; 2 MRC International Statistics and Epidemiology Group, Department of Infectious Disease Epidemiology, LSHTM, London, UK; 3 StatsDecide Analytics and Consulting Limited, Nairobi, Kenya

**Keywords:** Automated systematic review, Abstract screening, Latent Dirichlet Allocation, Topic modelling, Unsupervised learning

## Abstract

**Background:**

The importance of systematic reviews in collating and summarising available research output on a particular topic cannot be over-emphasized. However, initial screening of retrieved literature is significantly time and labour intensive. Attempts at automating parts of the systematic review process have been made with varying degree of success partly due to being domain-specific, requiring vendor-specific software or manually labelled training data.

Our primary objective was to develop statistical methodology for performing automated title and abstract screening for systematic reviews. Secondary objectives included (1) to retrospectively apply the automated screening methodology to previously manually screened systematic reviews and (2) to characterize the performance of the automated screening methodology scoring algorithm in a simulation study.

**Methods:**

We implemented a Latent Dirichlet Allocation-based topic model to derive representative topics from the retrieved documents’ title and abstract. The second step involves defining a score threshold for classifying the documents as relevant for full-text review or not. The score is derived based on a set of search keywords (often the database retrieval search terms). Two systematic review studies were retrospectively used to illustrate the methodology.

**Results:**

In one case study (helminth dataset), $69.83\%$ sensitivity compared to manual title and abstract screening was achieved. This is against a false positive rate of $22.63\%$. For the second case study (Wilson disease dataset), a sensitivity of $54.02\%$ and specificity of $67.03\%$ were achieved.

**Conclusions:**

Unsupervised title and abstract screening has the potential to reduce the workload involved in conducting systematic review. While sensitivity of the methodology on the tested data is low, approximately $70\%$ specificity was achieved. Users ought to keep in mind that potentially low sensitivity might occur. One approach to mitigate this might be to incorporate additional targeted search keywords such as the indexing databases terms into the search term copora. Moreover, automated screening can be used as an additional screener to the manual screeners.

**Supplementary Information:**

The online version contains supplementary material available at 10.1186/s13643-022-02163-4.

## Introduction

Over the years, the volume of published and unpublished literature has increased due to increased research interest and funding. To inform future research topics as well as avoid reinventing the wheel, there is need to review, collate and summarise available information on a particular research domain in a consistent manner. Systematic reviews are one of the popular and structured ways of evaluating existing literature. Well-designed and executed systematic reviews provide comprehensive assessment and act as a reliable summary of existing evidence for a research domain [1, 2]. Systematic reviews demand that the review process can be reproduced and is transparent in the steps taken to appraise existing literature. Systematic reviews have been used to appraise evidence in variety of research domains such as social and behavioural sciences [3], environment [4], education [5], health [6] and in business [7].

The Cochrane collaboration’s handbook provides methods and guidelines for conducting and reporting systematic reviews [1]. According to these guidelines, the key stages of a review include planning (protocol development and registration), conducting searches from databases based on an apriori*-*tested search strategy, managing retrieved articles which involved screening for article relevance, quality assessment, synthesising data and writing up of findings. While developing the protocol and conducting database searches may not be time and labour-consuming, screening of articles for relevance is both time and labour-intensive.

Often, screening documents for relevance in systematic reviews involves at least two reviewers who in the first stage, read titles and abstracts of documents retrieved from the database searches to assess relevance. The second stage involves reading full text of the subset of relevant documents from stage one to further assess relevance to the research topic [1, 8] —  a tedious process that consume substantial man-hours (estimated at up to two abstracts per minute for experienced reviewers). More importantly, at the abstract screening stage, there is often a big attrition rate with only a much smaller subset proceeding to full text review. For reviews on medical interventions or environmental topics, it is estimated that approximately 97% of the initially retrieved documents are considered irrelevant for further evaluation based on the initial title and abstract screening [9, 10]. Furthermore, the amount of time spent screening documents (based on title and abstract only) for relevance is estimated in the range of 10–20% of the total time it takes to plan and conduct a systematic review [10].

In particular, for extensively researched domains, the sheer volume of literature meeting the search criteria may be overwhelming. For instance, a study on completed reviews published on the Prospective Systematic Review Protocol Registry (PROSPERO) reported that database searches yielded between 27 and 92,020 documents [9]. Rather than manually screen such a huge collection of literature, innovative automated methods to speed-up the screening process may be beneficial [11–14].

There have been several attempts- both supervised and unsupervised- to automate the process of abstract screening in literature review — often involving text mining or active learning approaches [15–18]. Whereas both supervised and unsupervised learning of automation systems require preferably large training data, unsupervised learning methods do not require pre-labelled data for training. Therefore, they are more easily generalized to new domains potentially unseen in the training set. On the other hand, supervised learning algorithms learn the classification rules based on the training data set hence limiting their applicability to research domains they were trained on [19]. So far, results from existing automated screening algorithms estimate an average success rate between 30 and 70% in reducing the number of documents that have to be screened manually, albeit accompanied with some loss of potentially relevant studies [17, 18, 20].

Our primary objective was to develop statistical methodology for performing automated title and abstract screening for systematic reviews. Secondary objectives included (1) to retrospectively apply the automated screening methodology to previously manually screened systematic reviews and (2) to characterize the performance of the automated screening methodology scoring algorithm in a simulation study.

We propose a two-stage unsupervised approach based on topic modelling with Latent Dirichlet Allocation (LDA) [21] in combination with a “search keywords” corpus to automate screening of documents title and abstract. This way, we aim to overcome the main challenge of purely supervised methods: the need of pre-labelled training data is replaced by using the standard systematic review database search keywords, inclusion and exclusion criteria text. In the first stage, we apply LDA- an unsupervised topic modelling approach- to extract thematic areas (topics) expressed by the documents’ title and abstract. Once the topics are extracted, the second stage involves scoring the database search keywords, inclusion and exclusion criteria based on their word-topic probabilities. From this, a threshold score for the search keywords is defined which can subsequently be used to classify current and future documents as relevant or irrelevant for full text review.

While similar in spirit to most of the models proposed by Miwa et al. [16] and Mo et al. [22] who applied LDA as a summary measure of the documents’ content, our approach differs from these authors’ in that, we propose a completely unsupervised approach. The active learning approach by Miwa et al. [16] requires an initial set of manually labelled documents from which the algorithm actively learns to classify subsequent documents. Similarly, Mo et al. [22] uses the LDA-derived topics as input for a Support Vector Machine classification- a supervised learning algorithm.

An approach by Li et al. [23] is more similar to our work in two ways: first, it is a purely unsupervised classification approach. Secondly, their input to the classification algorithm includes both the title and abstract text and a user-defined semantics (keyword) although they additionally use the index term list derived from indexing databases such as MEDLINE and EMBASE. However, their work differs from ours mainly on how the relevance score and threshold is determined. The keywords and index term relevance are determined with a Lucene score [24] and their approach to utilizing the LDA-derived metrics for relevance scoring is also different. Our view is that a simple percentile-based approach to relevance scoring approach would be preferred as it is easier to implement in classical statistical software such as R and Statistical Analysis Software (SAS) since this step only involves an arithmetic calculation. On the other hand, there is potentially, strength in their use of additional input data from indexing databases to enrich the document relevance classification- a component that may be considered in our subsequent work.

To illustrate the utility of the proposed unsupervised title and abstract screening for systematic reviews, data from two completed systematic reviews will be used. A systematic review assessing the effect of helminths on vaccines response (Case study 1: the helminths data) — a planned systematic review for which full-text review of documents has been completed [25] and from a published systematic review on the effectiveness of therapies for Wilson disease (Case study 2: Wilson disease data) [26] will be analysed. For both case studies, information on initial records identified through database search and results after manual title and abstract screening is available. Ultimately, we will compare the results of our methodology with the manual screening results. Further, we performed a simulation study to evaluate the impact of composition and size of the training data as well as that of the scoring threshold on classification.

The article is arranged as follows: first, we describe the two case studies in details. Subsequently, the methodology for both topic extraction and document scoring is described, followed by results of its application to the two case studies. The simulation study settings and results are then described followed by a concluding section.

## Data

Two sets of data are required: (1) Document corpus *D*: a text file in a standard format (such as bibliography files from bibliography management software) containing the collection of document title and abstract for all articles identified based on database search. (2) Search keywords data *S*: a text file detailing the database search terms, inclusion and exclusion criteria.

### Case study 1: the helminths data

A systematic review on the effect of helminths on vaccine responses in human participants was performed. Records were retrieved from several databases using search terms on helminth and vaccine types. The protocol for the review was prospectively registered in PROSPERO [25]. The document corpus comprises of 1318 documents which upon screening by two reviewers (based on document title and abstract), 116 were considered relevant for full-text screening — indicating an attrition rate of $91\%$. Only 28 of these documents were used in the final systematic review — a success rate of $2\%$ from the initially retrieved documents. For each document in the corpus, both the title and abstract text were combined into one string. The search keywords data, *S*, comprised of unique words derived from the search strategy section of the systematic review protocol [25]. This study is used to illustrate the methodology development and application.

### Case study 2: Wilson disease data

The second case study involved data from an already published systematic review on the effectiveness of therapies for Wilson disease [26]. The data and details of the document screening process were accessed from a data repository [27]. The document corpus comprised of 3453 records which upon title and abstract screening by two reviewers, only 174 were considered relevant for full-text review — an attrition rate of $95\%$. Subsequently, after a manual full-text review, only 26 of these documents were included in the final systematic review—  a success rate of less than $1\%$ compared to initially retrieved documents.

From the search strategy provided as an appendix by Appenzeller-Herzog et al. [26], we processed the text in search strategies $1-10$ by splitting the combined text into individual words, removed stop words, duplicates and punctuation resulting in 99 unique search keywords corpus *S*.

### Data pre-processing

Prior to estimation, standard textual data prepossessing steps are necessary. For instance, removal of frequent or non-specific words (stop words: and, or, the etc.), punctuation and numbers. We further cleaned up extra white spaces in words and filtered words to have at least two characters (can be used to filter out chemical symbols or other abbreviations as may be deemed necessary). These options were passed to the DocumentTermMatrix() function of the topicmodels R package although similar results can be achieved using tidytext R package (with some effort).

A critical step in the methodology presented in this manuscript is stemming of words to extract the root for each word [28]. This is performed for both the document corpus *D* and the search keywords corpus *S* so that words with the same root appearing in both datasets can be matched.

## Methods

This section describes the topic modelling methodology and subsequent scoring algorithm used for document labelling as well as the simulation study setting.

### Latent Dirichlet Allocation

Consider a corpus *D* whose elements are the individual title and abstract text retrieved from initial database search. For this corpus, the set *V* of unique words defines the vocabulary vector of the corpus. For each word in the vocabulary, the frequency of its occurrence in each document is known (term frequency). Latent Dirichlet Allocation (LDA) — being a generative probabilistic model — assumes that a document $\boldsymbol{W_m}$, $m=1, 2, \ldots , M$ such that $\{\boldsymbol{W_1}, \boldsymbol{W_2}, \ldots \boldsymbol{W_M}\} \in D$ comprises of *N* words (possibly a subset $N \subset V$ ) from the vocabulary. That is, $\boldsymbol{W_m}=(w_1, w_2, \ldots , w_N)$ is a bag of words from the vocabulary where there no particular ordering of the words.

The generative process for each document $\boldsymbol{W}=\{\boldsymbol{W_1}, \boldsymbol{W_2}\ldots \boldsymbol{W_M}\}$ proceeds as follows:

1. Choose $N \sim Poisson(\xi )$: number of words comprising a document, where $\xi$ is a hyperparameter.
2. Choose $\theta \sim Dir(\alpha )$: proportion of each topic in a document, where $\alpha$ is a hyperparameter.
3. For each of the *N* words $w _n$:
  - Choose a topic $$z_n \sim Multinomial(\theta )$$.
  - Choose a word $w _n$ from $p( w _n|z_n, \beta )$: a multinomial probability conditioned on the topic $z_n$.

The quantities of interest are the word-topic probability matrix $\boldsymbol{\beta }$ — denoting the distribution of words across topics $Z={z_1, z_2, \ldots , z_K}$ — and the document-topic probability matrix (denoted as $\boldsymbol{\gamma }$). Bayesian inference [29] with Gibbs sampling is then used to estimate the posterior probabilities of the quantities of interest [21, 30].

Current implementation of LDA in R topicmodels package requires a user to specify the number of topics *K* to extract as a fixed parameter. A guesstimate of the plausible number of topics given a document corpus *D* may be obtained using ldatuning R package [31].

The primary output of the LDA model comprises of two matrices: (1) the posterior word-topic probability matrix $\boldsymbol{\beta }$ is a $V\times K$ denoting the posterior probability of each word given a topic and (2) the posterior document-topic probability matrix $\boldsymbol{\gamma }$ is an $M \times K$ matrix denoting the topic-mixture composition for each document.

### Search keywords scoring

The matrix $\boldsymbol{\beta }$ is used to define a score for each term in the search keywords corpus *S* for each of the derived LDA topics. For each topic $Z_k \in \boldsymbol{Z}$, where $k=1, 2, \ldots K$, define a matrix $\boldsymbol{T}$ as the rows in $\boldsymbol{\beta }$ matching the words in *S*. Note that in some instances, some words in *S* may not appear in any of the documents hence missing a match in $\boldsymbol{\beta }$. Such words are excluded from further processing. Subsequently, calculate the joint search keywords probability for each topic as the column sum of $\boldsymbol{T}$ (LDA assumes independence of words within a topic) resulting in a $1\times K$ vector of search keywords scores. The distribution of search keywords scores can be visualized in a histogram. The next step involves determining a score threshold $h_{i\%}$ which we specify as a percentile $P_{i\%}$ of the score distribution such that $1-P_{i\%}$ of the scores are larger than the threshhold $h_{i\%}$. An optimal choice of $P_{i\%}$ is explored in a simulation study. The subset $\boldsymbol{R} \subset \boldsymbol{Z}$ of topics with search keywords score greater than or equal to $h_{i\%}$ is then used for determining the document relevance score $\varvec{U}_{h_{i\%}}$ (sum of scores greater than or equal to $h_{i\%}$) and the document-keywords score.

### Document-keywords score

For each document, calculate the weighted sum of the search keywords probability in the $\boldsymbol{R}$ topics. The weight is defined as the frequency with which each search keyword occurs in a given document. Subsequently, a document is considered relevant for full-text screening if its search keywords score is larger than or equal to the document relevance score threshold $\boldsymbol{U}_{h_{i\%}}$.

### Simulation study

We performed a simulation study to evaluate several aspects of the unsupervised classification algorithm:

1. The proportion of relevant documents in the training corpus. Given the high attrition rate of retrieved documents after a manual title and abstract screening, it is of interest to evaluate the performance of the algorithm even when few or no relevant documents are in the training corpus.
2. The role of the size of the training corpus — number of documents used to define the relevance score.
3. The number of LDA topics extracted. LDA requires a user to specify the number of topics to extract and although some metrics may be available to guide on an optimal number of topics, we evaluate the impact of this choice on the final classification.

Note that the helminth data was used to evaluate these three objectives in a simulation by sampling some documents as training set and using the remaining documents as a test set. Based on the results of the simulation study, optimal choices were made for the analysis of the Wilson disease data to sort of validate these simulation findings with a new dataset. In the following section, we describe the parameters used in the simulation study.

#### Proportion of relevant documents

Relevance (for full-text review based on manual title and abstract screening) of each document in the helminth dataset was known since these had been manually evaluated previously. We selected a proportion $(0\%, 25\%, 50\%, 75\%, 100\%)$ of the relevant documents into the training corpus.

#### The size of the training corpus

In routine use, once a big enough collection of documents has been retrieved, the algorithm is applied to all available documents at once. Hence, no splitting of the documents corpus into training and test set is needed. However, to evaluate the possible impact of performing title and abstract screening when only a subset of the potentially available literature is available, we sampled $75\%$ of the available documents (while maintaining the proportion of relevant documents as above) as the training set. Note that the remaining $25\%$ of the documents were used as the test set from which performance metrics were computed.

#### The number of LDA topics to model

For the helminth data, standard metrics [32–34] suggested 20 topics as an optimal choice of topics to model. We evaluated a range of topics from few to much higher number of topics (2, 10, 20, 40, 100).

The combination of the above three aspects was simultaneously evaluated in 1000 simulations. In each iteration, a percentile $P_{i\%}=0\%,\, 50\%,\, 80\%,$ $85\%,\, 90\%,\,$ $95\%,\, 100\%$ of the search keywords score distribution is computed (refer to the methodology section).

The simulation performance is evaluated by computing performance metrics such as the average true positive rate (sensitivity), false negative rate, false positive rate and the true negative rate (specificity).

## Results

In this section, we first present the results based on the helminths dataset. In this dataset, relevance (for full-text review) label based on manual screening was available. Hence, we can evaluate the unsupervised model’s performance in retrieving the manually assigned labels. Note that in routine use, the relevance label of documents is not available upfront. Our methodology seeks to automate generation of a relevance label for retrieved documents.

### The helminths data

#### Standard LDA output

**Fig. 1:**
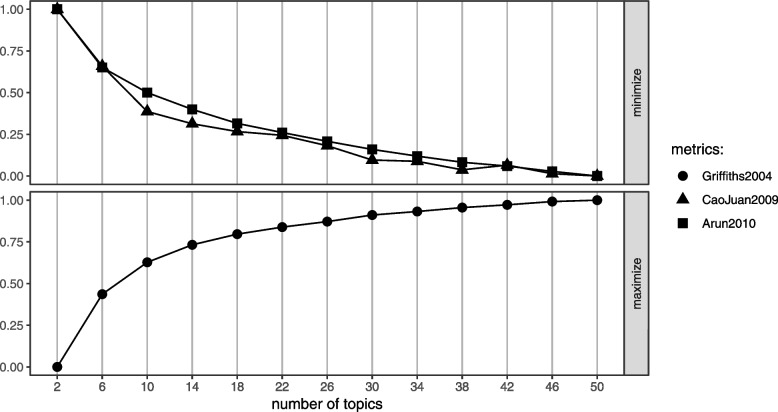
The helminths data: grid search of number of LDA topics. Both Aruna2010 and CaoJuan2009 metrics are based on minimization while Griffiths2004 is a metric based on maximization of the corresponding algorithm. *Y*-axis: Normalized measure of performance

To determine the number of topics to model with LDA, a grid search of topics between 2 and 50 was performed using the FindTopicNumber() function [31]. The function computes a normalized score for two metrics based on minimization of an objective function and one based on a maximization algorithm. From the grid of topics explored, we note that initially, the metric score decreases (or increases for maximization algorithm) with increasing number of topics. The change in metric score reduces substantially as an optimal range of topics is explored. Figure 1 shows that all the metrics reach a plateau in the range of 18 to 30 topics. For subsequent analysis, we model 20 topics using Gibbs sampling algorithm executed with a burn-in of 100, 3 chains and 2000 iterations.

**Fig. 2:**
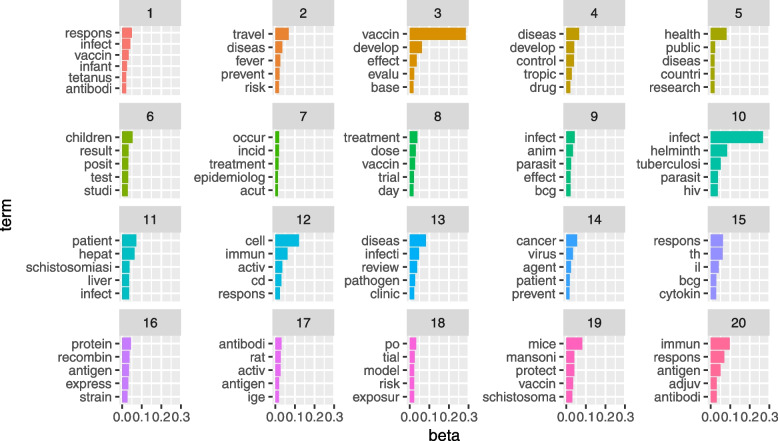
The helminths data: Word-topic probability matrix $\varvec{\beta }$. Top 5 words (in their root form) for each of the 20 topics. *X*-axis: posterior word-topic probability

**Fig. 3:**
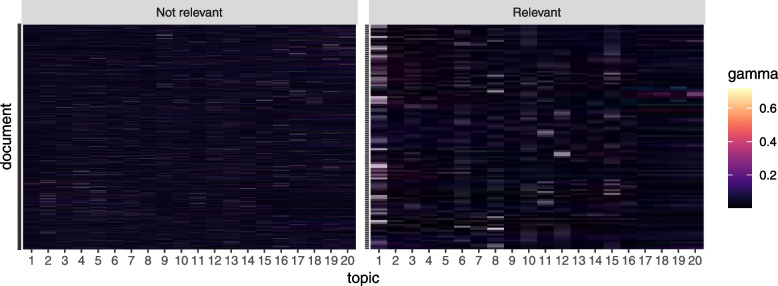
The helminths data: document-topic probability matrix $\varvec{\gamma }$ for the full corpus. *X*-axis: posterior document-topic probability. *Y*-axis: individual documents in the corpus. The panels denote documents considered relevant (or not) for full-text screening based on manual evaluation

Figure 2 shows the posterior word-topic probability for the top 5 words from each topic (based on their posterior word-topic probability, $\varvec{\beta }$). Often, the main themes of each topic can be inferred from the top words in a topic. For instance, topic 5 seems to detail public health research in general, while topics 12,  17 and 20 seems to address humoral and T cell-mediated immune response. In LDA, words are considered to be exchangeable (“bag-of-word” assumption) and may appear in multiple topics with varying probability. Moreover, a document may comprise of only a subset of topics as shown in Fig. 3. Topics 1 and 8 were dominant in documents selected as relevant for full-text screening based on manual title and abstract screening by two reviewers, while topics $2-5, 16-20$ had the least contribution in documents selected as relevant for further screening. This is expected since topics 1 and 8 mainly contain words related to helminths infection, vaccination and treatment (see word-clouds for each topic in Additional file 1).

#### Search keywords scoring

**Fig. 4:**
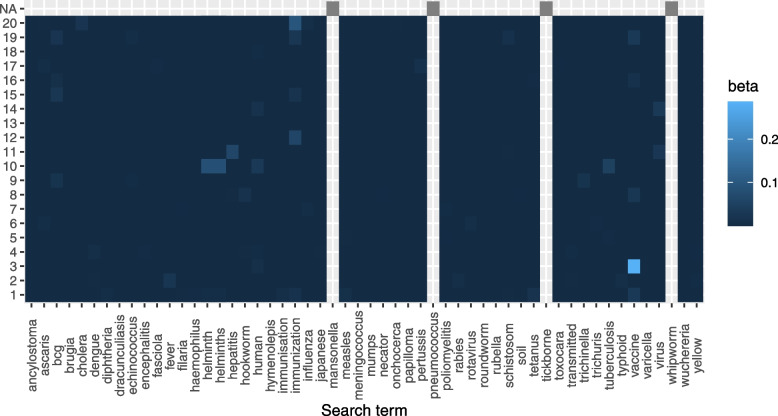
The helminths data: search keywords posterior word-topic probability $\varvec{\beta }$. Fill colour gradient: posterior word-topic probability. X-axis: individual search keywords in the corpus. Y-axis: Topics. NA: search keyword did not occur in documents

**Fig. 5:**
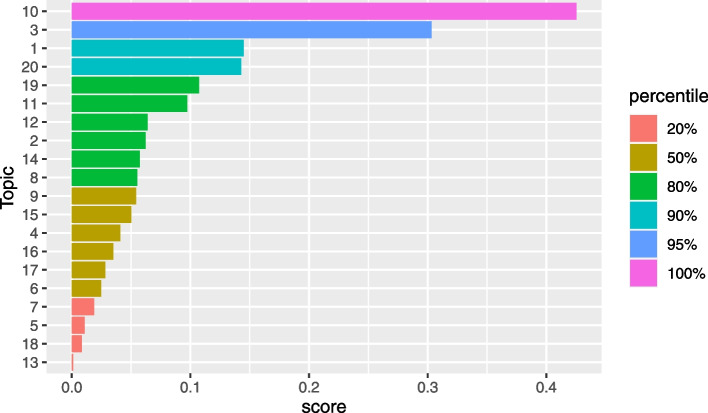
The helminths data: search keywords sum of word-topic probability $\varvec{\beta }$. *X*-axis: posterior word-topic probability. *Y*-axis: individual search keywords in the corpus. Colour: corresponding percentile (of the sum of search term scores) the topics cover

The search keywords corpus was pre-processed the same way as the title and abstract corpus and comprised of 54 words. For each LDA topic, the posterior probability matrix of the search keywords $\varvec{T}$ was extracted from the word-topic posterior probability matrix $\varvec{\beta }$. Figure 4 shows the posterior word-topic probability for the search keywords across all topics. Search keywords such as helminths, hepatitis, immunization and vaccines had a high posterior topic probability, while search keywords such as mansonella, pneumococcus, whipworm and tickborne did not occur in the retrieved documents. Such search terms that did not appear in any of the documents are excluded from further analysis since they do not contribute to the classification score. Figure 5 shows a histogram of the sum of posterior probabilities of all search words per topic, from which, topics 1,  3,  10 and 20 contain top $20\%$ of the search term sum of scores.

To derive the relevance threshold, we specify a percentile and select the topics with a sum of word-topic score above that percentile as useful for selecting documents relevant for full-text search. For instance, the topics above the $P_{80\%}$ percentile include 1,  3,  10 and 20. Based on these four topics, the sum of scores $\varvec{U}_{h_i\%}=1.01630$ (which is the sum-total of topics 1, 3, 10 and 20 score values as depicted in the X-axis of Fig. 5). is used to determine documents’ relevance.

#### Predicting document relevance

To determine the relevance of a document for full-text screening, the subset $\varvec{R}$ of relevant topics is explored. Given a document, for these topics in $\varvec{R}$, the weighted posterior sum of word-topic probability for the search keywords appearing in the document is calculated whereby, the weights are the frequency of occurrence of each search keyword in a document. Note that there is no restriction imposed on the weights to sum to one. Hence, if specific search keywords are extensively used in a document, the resulting score is higher compared to a document where the search keywords are barely mentioned.

For each document, if the weighted sum of word-topic probabilities (for the search terms appearing in the document) is higher than the preset threshold $\varvec{U}_{h_i\%}$ at the selected score percentile, the document is considered relevant for full-text screening. Classification performance of the unsupervised LDA algorithm on the helminths title and abstracts data is summarised in Table 1.

**Table 1:** The helminths data: classification performance of the unsupervised LDA algorithm based on documents title and abstract. The relevance threshold is calculated based on the 80th percentile of the search keywords score

		Automated prediction		
		Not relevant	Relevant	Total
**Manual assignment title/abstract**	**Not relevant**	930	272	1202
	**Relevant**	35	81	116
	**Total**	965	353	1318

For this data, the classification algorithm had a sensitivity of $69.83\%$ based on unsupervised title and abstracts screening. Note that, although the false positive rate is $22.63\%$, this is still significantly fewer documents to perform a full-text review on compared to all the true negatives identified by the algorithm that would no longer require manual review (specificity of $77.37\%$). As expected, there is a trade-off in that, some truly relevant documents may be classified as irrelevant (false negative rate of $30.17\%$) for full-text screening based on automated title and abstract screening. On the other hand, it is possible to re-run the algorithm on the subset of documents initially flagged as irrelevant to potentially identify more relevant documents.

We further compare the proportion of documents classified as relevant for full-text screening by the unsupervised title and abstract screening that were truly relevant for meta-analysis after manual full-text screening. Of the 116 documents that were manually flagged as relevant for full-text screening, 81 of them were also flagged as relevant for full-text screening by the unsupervised algorithm. After manual full-text review, only 28 out of the 116 documents were used for subsequent systematic review steps. 18 out of these 28 documents were already flagged as relevant for full-text screening by the automated algorithm as shown in Table 2.

**Table 2:** The helminths data: Manual title and abstract as well as full-text classification versus automated title and abstract screening classification. Zero imputation: since documents were excluded from manual full-text screening, they could not be found relevant after manual full-text screening

		Manual full-text	Automated prediction		
		screening	Not relevant	Relevant	Total
**Manual assignment based on title and abstract screening**	**Not relevant**	**Not relevant**	930	272	1202
		**Relevant**	0	0	0
	**Relevant**	**Not relevant**	25	63	88
		**Relevant**	10	18	28
	**Total**		965	353	1318

### Simulation study

**Fig. 6:**
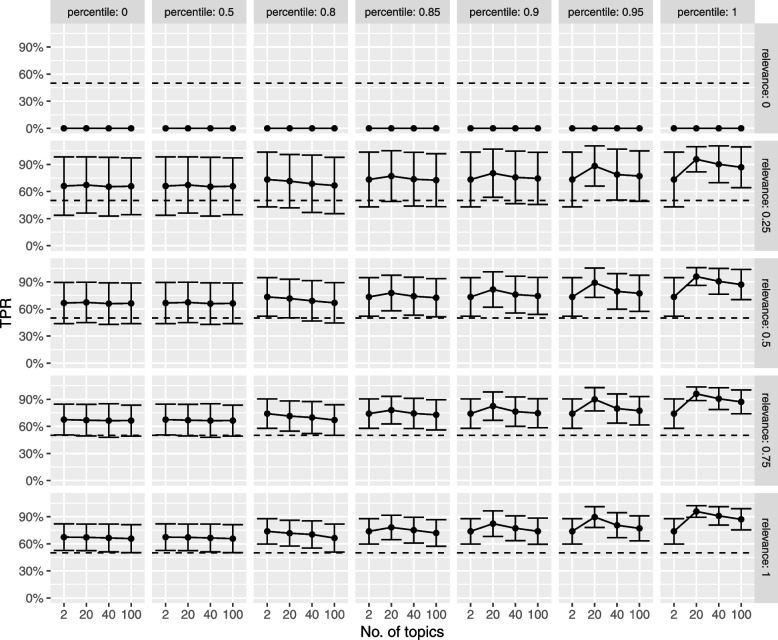
Simulation study: average sensitivity results. *X*-axis: number of LDA topics. TPR, true positive rate. Rows: proportion of relevant documents included in the simulation dataset. Columns: percentile used to compute the relevance threshold. Solid circles: average TPR. Error bars: 95% confidence interval. Horizontal dashed line: 50% TPR

The average true positive rate (sensitivity) based on 1000 resamples of the helminth dataset is summarised in Fig. 6. Note that 75% of the proportion of relevant documents (and 75% of the documents originally flagged as not relevant) were included in the training superset with the remaining 25% of each category being used as the test superset for which sampling proportions used to compute the metrics shown in Fig. 6 were derived.

Overall, modelling an optimal number of LDA topics (20 for the helminth dataset) provides the best sensitivity. Moreover, in determining the relevance threshold for scoring new documents, a percentile above $80\%$ results in a higher true positive rate. In particular, the 85th percentile has a lower bound of the $95\%$ confidence interval above $50\%$. As expected, sensitivity increases with the proportion of relevant documents included in the training set.

**Fig. 7:**
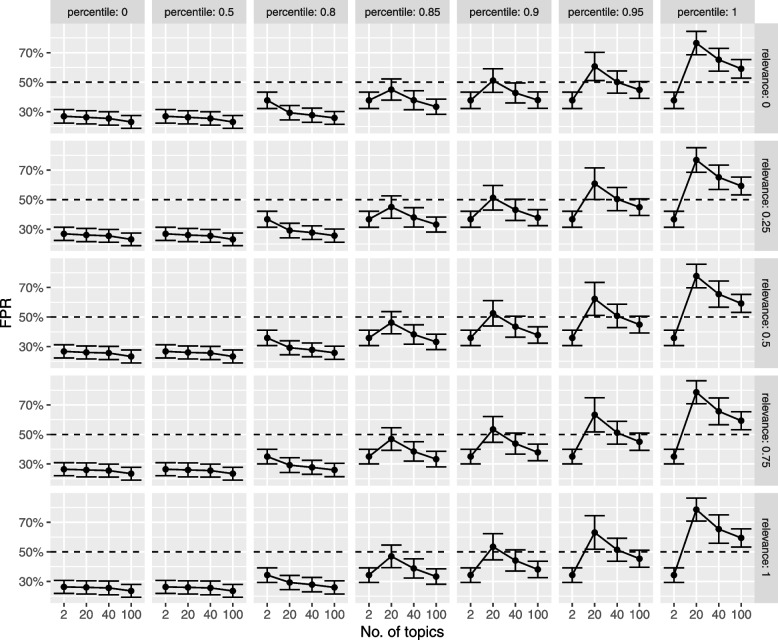
Simulation study: average false positive rate results. *X*-axis: number of LDA topics. FPR, false positive rate. Rows: proportion of relevant documents included in the simulation dataset. Columns: percentile used to compute the relevance threshold. Solid circles: average FPR. Error bars: 95% confidence interval. Horizontal dashed line: 50% FPR

From Fig. 7, the optimal scenario in terms of sensitivity has an upper bound of the $95\%$ confidence interval for the false positive rate below $60\%$. In this case, the false positive rate is quite comparable to that observed when no relevant documents are included in the training set.

### External validation: Wilson case study

**Fig. 8:**
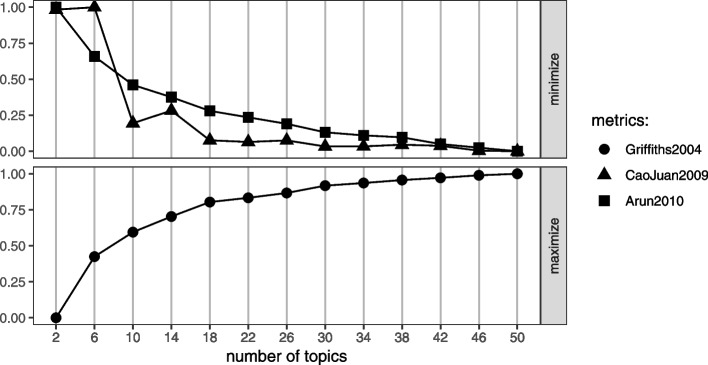
The Wilson data: grid search of number of LDA topics. Both Aruna2010 and CaoJuan2009 metrics are based on minimization while Griffiths2004 is a metric based on maximization of the corresponding algorithm. *Y*-axis: Normalized measure of performance

For this study, guided by the topics selection metrics shown in Fig. 8 and the conclusions of the simulation study above, we modelled 35 topics using LDA. From this model, the word-topic probability distribution matrix $\varvec{\beta }$ as well as the document-topic probability distribution matrix $\varvec{\gamma }$ were extracted and used to further score the search keywords for relevance.

**Fig. 9:**
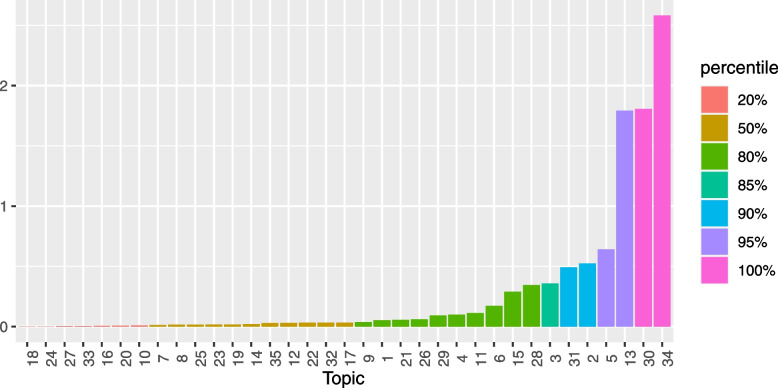
The Wilson data: search keywords sum of word-topic probability $\varvec{\beta }$. *X*-axis: posterior word-topic probability. *Y*-axis: individual search keywords in the corpus

The distribution of the corresponding scores for the chosen search keywords is shown in Fig. 9. From these scores, given a desired percentile $P_{85\%}$, topics $2,\, 5,\,13,\, 30,\, 31$ and 34 are chosen for document classification. For this study, the unsupervised classification algorithm had a sensitivity of $54.02\%$ and specificity of $67.03\%$ as derived from Table 3.

**Table 3:** The Wilson data: Manual full-text classification versus automated title and abstract screening classification

		Automated prediction		
		Not relevant	Relevant	Total
**Manual assignment title/abstract**	**Not relevant**	2198	1081	3279
	**Relevant**	80	94	174
	**Total**	2278	1175	3453

**Fig. 10:**
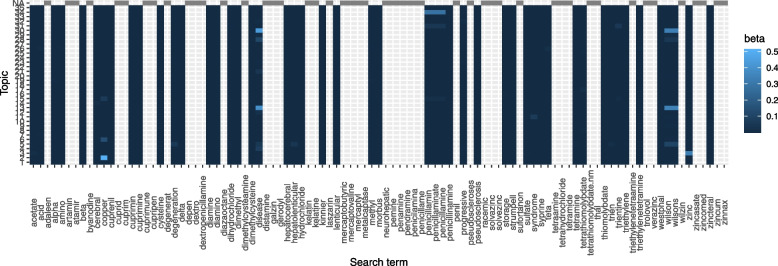
The Wilson data: search keywords sum of word-topic probability $\varvec{\beta }$. *X*-axis: posterior word-topic probability. *Y*-axis: individual search keywords in the corpus. Colour: corresponding percentile (of the sum of search term scores) the topics cover

Table 4 evaluates the proportion of documents that were considered relevant after manual full-text screening versus whether the automated abstract and titles screening would have selected them for full-text screening. We note that with the automated unsupervised learning approach, we fail to capture 15 out of the 26 documents selected as relevant for systematic review after manual full-text screening. This may partially be attributed to the choice of search keywords used to define the classification threshold. As shown in Fig. 10, most of these search keywords did not occur (or had very low word-topic probability) in title and abstracts of the documents since they were mostly chemical names. While these terms may appear more frequently in the full-text of the documents, they are very specific and may occur less in abstracts and titles. For this reason, it might be advisable to define search terms that describe the problem of interest in more general terms that might occur more frequently in document titles and abstracts.

**Table 4:** The Wilson data: Manual title and abstract as well as full-text classification versus automated title and abstract screening classification

		Manual full-text	Automated prediction		
		screening	Not relevant	Relevant	Total
**Manual assignment assignment title and abstract screening**	**Not relevant**	**Not relevant**	2276	1003	3279
		**Relevant**	0	0	0
	**Relevant**	**Not relevant**	64	84	148
		**Relevant**	15	11	26
	**Total**		2355	1098	3453

## Discussion

In this manuscript, we implemented a two-stage classification algorithm for automating documents title and abstract screening — a significant and time-consuming initial step while conducting systematic reviews. The initial automation step involves deriving thematic areas covered by the documents by fitting a Latent Dirichlet Allocation natural language processing model to retrieved documents’ title and abstract corpus. The second step involves scoring a pre-defined set of keywords from which document classification is performed.

As implemented, the methodology is easily generalizable to any research domain as no pre-labelled training data is required. However, the choice of the contents of the search keywords corpus has an influence on the algorithm’s classification performance. Classically, systematic reviews have a clearly laid out search strategy, inclusion and exclusion criteria, which presents an obvious choice for the search keywords corpus. Nevertheless, the search keywords text should be descriptive enough such that, most of these keywords naturally occur in the title and abstract of documents being retrieved. For instance, chemical names and symbols or domain-specific abbreviations may occur less-frequently in documents title and abstract thus rendering such words less efficient in scoring and classifying the respective documents. If domain-specific keywords such as those indexed by databases such as the Medical Subject Headings (MeSH) are available, they could be used to enrich the search keywords corpus.

**Fig. 11:**
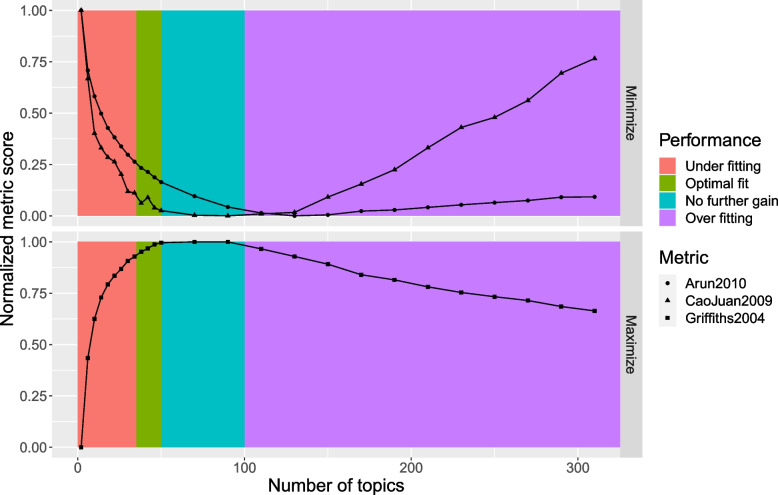
Example scree plot: Determining an optimal number of topics to model. *X*-axis: Number of topics. *Y*-axis: Normalized metric score. Colour bands: interpretation of the metric scores for the corresponding number of topics

The choice of LDA topics to extract may impact the classification algorithm’s performance. While tools are available to guide on plausible number of LDA topics to extract, they are often computer intensive and not explicit on the exact number of topics to model. However, the time invested in searching for plausible number of topics to model may be worthwhile considering the impact this parameter has on classification. As a rule of thumb, the number of topics corresponding to the elbow of a scree-plot of the normalized scores versus number of topics may be used as an optimal choice of topics. When there is no clear change point in the scree plot, this might be an indicator that no clear thematic areas are extracted based on the current LDA settings. Too many topics may overfit the documents corpora resulting in poor classification performance. An illustrative hypothetical example is provided in Fig. 11. Too many topics are an indication of over fitting. However, it is worthy to note that an optimal number of topics does not necessarily translate to human-coherent topics. If desirable to also have a measure of topic quality, additional topic quality assessment may be performed using metrics such as coherence and perplexity scores.

Fitting of LDA models is not trivial. To begin with, unlike datasets routinely used in statistical analyses that are often in tabular format, input data for LDA is often derived from reference management platforms hence effort is required to import them into statistical software and structure. To this end, some basic skills in natural language processing may be necessary especially to aid in cleaning up and exploring the resulting dataset. Besides, there are various R packages that can be used to fit LDA models which may vary to some extent in their capabilities and requirements in terms of acceptable data structure. We illustrated the use of topicmodels R package in fitting LDA models utilizing a term-frequency weighing. If a term frequency inverse document weighing is required, this may not be trivial to implement for some users. However, once the data processing hurdle is overcome, out of the box, the fitting of LDA models is straight forward. From the fitted LDA model, calculation of the score threshold involves simple arithmetic computations and is not complex to average users.

The benefits of modelling an optimal number of LDA topics is critical to good performance of the automated classification and cannot be over-emphasized. By extracting an optimal number of topics, the resulting search keywords score distribution would ideally give the most weight to a few topics. From the distribution, as a rule of thumb, we postulate that the 80th percentile would optimally provide the best classification. Hence, topics covering this percentile can be used to compute the relevance threshold score for subsequent document classification. More work is planned to further explore this in different settings in future.

The intent of automating the title and abstract screening step is to reduce the volume of documents that are manually reviewed and subsequently found inappropriate for full-text review. While conducting systematic reviews, document attrition rates are high with approximately 5% of initially retrieved documents being considered relevant for the final analysis. Thus, an automated system with high sensitivity is required as a minimum and simultaneous high specificity would be desirable. With the unsupervised approach presented here, sensitivity of at least $54\%$ was observed for both case studies. Potentially, higher sensitivity might be obtained by (1) critically assessing the content of the search keywords corpus, the impact of the vocabulary size in this corpus and potentially enriching it with domain-specific keywords that have a higher frequency of occurrence in documents titles and abstracts and (2) re-evaluating the relevance score calculation and possibly redefine the classification from a binary (relevant/irrelevant) labelling to a probability score of relevance.

Once documents are labelled by the automated system, users have the option to review a subset of those documents initially flagged as irrelevant for full-text review since it may contain relevant documents that are incorrectly labelled (false negatives). We propose that all the documents flagged as irrelevant for full-text review are used as a new corpus for which the algorithm is re-run at least once. This way, the relevance threshold is recalculated and additional relevant documents may be identified. Note that the unsupervised model’s false positive rate in the absence of truly relevant documents was approximately $30\%$ in the simulation study. Therefore, a balance between additional re-runs to improve on overall sensitivity and specificity and the additional workload of manually reviewing irrelevant documents must be struck.

## Conclusions

Overall, fully unsupervised screening of titles and abstracts in systematic reviews seems feasible. The combination of LDA and a well-defined relevance score has the advantage that no additional pre-labelled data is required for classification. Further, the current approach is easily generalizable to new domains since the model inputs are standard systematic review datasets. For that reason, there is no extra data collection and labelling effort required from the user to implement the methodology.

The proposed approach uses simple and standard natural language processing tools available in open-source statistics software (an example RMarkdown workflow is provided in Additional file 2). Therefore, users can easily develop an analysis pipeline without requiring additional commercial automation tools as is the case with some currently available automated screening tools. There is still potential to improve on sensitivity and specificity of the unsupervised screening model. To this end, future efforts will focus on the role of search keywords and how they can be best refined to improve performance.

## Supplementary Information

**Additional file 1.** Zip file containing visualization of the words comprising derived LDA topics for the helminths dataset.

**Additional file 2.** The file can be viewed in any browser and provides the relevant R code for the methodology as applied to the helminths case-study.

## Data Availability

The Wilson dataset is publicly available as described in the data section and accompanying references. The workflow and corresponding statistical software for analysing this dataset is provided as supplementary materials.

## References

[1] JPT H, J T, J C, M C, T L, MJ P, et al., editors. Cochrane Handbook for Systematic Reviews of Interventions. 2nd ed. Chichester: Wiley; 2019.

[2] Clarke J (2011). What is a systematic review?. Evid-Based Nurs..

[3] Kwon HR, Silva EA (2019). Mapping the Landscape of Behavioral Theories: Systematic Literature Review. J Plan Lit..

[4] Bilotta GS, Milner AM, Boyd I (2014). On the use of systematic reviews to inform environmental policies. Environ Sci Policy..

[5] Zawacki-Richter O, Kerres M, Bedenlier S, Bond M, Buntins K, editors. Systematic Reviews in Educational Research. USA: Springer Fachmedien Wiesbaden; 2020. 10.1007/978-3-658-27602-7.

[6] Johnson BT, Low RE, LaCroix JM (2013). Systematic Reviews to Support Evidence-based Medicine (2nd edition) by Khalid Khan, Regina Kunz, Jos Kleijnen and Gerd Antes: A Review. Res Synth Methods.

[7] Konstantinidis I, Siaminos G, Timplalexis C, Zervas P, Peristeras V, Decker S. Blockchain for Business Applications: A Systematic Literature Review. In: Business Information Systems. Cham: Springer International Publishing; 2018. p. 384–399. 10.1007/978-3-319-93931-5_28.

[8] Edwards P, Clarke M, DiGuiseppi C, Pratap S, Roberts I, Wentz R. Identification of randomized controlled trials in systematic reviews: accuracy and reliability of screening records. Stat Med. 2002;21(11):1635–40. 10.1002/sim.1190. https://onlinelibrary.wiley.com/doi/abs/10.1002/sim.1190 10.1002/sim.1190 12111924

[9] Borah R, Brown AW, Capers PL, Kaiser KA. Analysis of the time and workers needed to conduct systematic reviews of medical interventions using data from the PROSPERO registry. BMJ Open. 2017;7(2). 10.1136/bmjopen-2016-012545. https://bmjopen.bmj.com/content/7/2/e012545. 10.1136/bmjopen-2016-012545 PMC5337708 28242767

[10] Haddaway NR, Westgate MJ. Predicting the time needed for environmental systematic reviews and systematic maps. Conserv Biol. 2019;33(2):434–43. 10.1111/cobi.13231. https://conbio.onlinelibrary.wiley.com/doi/abs/10.1111/cobi.13231. 10.1111/cobi.13231 30285277

[11] Tsafnat G, Glasziou P, Choong MK, Dunn A, Galgani F, Coiera E. Systematic review automation technologies. Syst Rev. 2014;3(1). 10.1186/2046-4053-3-74. 10.1186/2046-4053-3-74 PMC4100748 25005128

[12] Olofsson H, Brolund A, Hellberg C, Silverstein R, Stenström K, Österberg M, et al. Can abstract screening workload be reduced using text mining? User experiences of the tool Rayyan. Res Synth Methods. 2017;8(3):275–80. 10.1002/jrsm.1237. https://onlinelibrary.wiley.com/doi/abs/10.1002/jrsm.1237. 10.1002/jrsm.1237 28374510

[13] Beller E, , Clark J, Tsafnat G, Adams C, Diehl H, et al. Making progress with the automation of systematic reviews: principles of the International Collaboration for the Automation of Systematic Reviews (ICASR). Syst Rev. 2018;7(1). 10.1186/s13643-018-0740-7. 10.1186/s13643-018-0740-7 PMC5960503 29778096

[14] Marshall IJ, Wallace BC. Toward systematic review automation: a practical guide to using machine learning tools in research synthesis. Syst Rev. 2019;8(1). 10.1186/s13643-019-1074-9. 10.1186/s13643-019-1074-9 PMC6621996 31296265

[15] Feng L, Chiam YK, Lo SK. Text-Mining Techniques and Tools for Systematic Literature Reviews: A Systematic Literature Review. In: 2017 24th Asia-Pacific Software Engineering Conference (APSEC). 2017. p. 41–50. 10.1109/APSEC.2017.10.

[16] Miwa M, Thomas J, O’Mara-Eves A, Ananiadou S (2014). Reducing systematic review workload through certainty-based screening. J Biomed Inform..

[17] Wallace BC, Trikalinos TA, Lau J, Brodley C, Schmid CH. Semi-automated screening of biomedical citations for systematic reviews. BMC Bioinformatics. 2010;11(1). 10.1186/1471-2105-11-55. 10.1186/1471-2105-11-55 PMC2824679 20102628

[18] O’Mara-Eves A, Thomas J, McNaught J, Miwa M, Ananiadou S. Using text mining for study identification in systematic reviews: a systematic review of current approaches. Syst Rev. 2015;4(1). 10.1186/2046-4053-4-5. 10.1186/2046-4053-4-5 PMC4320539 25588314

[19] Berry MW, Mohamed A, Yap BW, editors. Supervised and Unsupervised Learning for Data Science. Springer International Publishing; 2020. 10.1007/978-3-030-22475-2.

[20] Cohen AM, Hersh WR, Peterson K, Yen PY (2006). Reducing Workload in Systematic Review Preparation Using Automated Citation Classification. J Am Med Inform Assoc..

[21] Blei DM, Ng AY, Jordan MI. Latent Dirichlet Allocation. J Mach Learn Res. 2003;3(null):993–1022.

[22] Mo Y, Kontonatsios G, Ananiadou S. Supporting systematic reviews using LDA-based document representations. Syst Rev. 2015;4(1). 10.1186/s13643-015-0117-0. 10.1186/s13643-015-0117-0 PMC4662004 26612232

[23] Li D, Wang Z, Wang L, Sohn S, Shen F, Murad MH, et al. A Text-Mining Framework for Supporting Systematic Reviews. Am J Inf Manag. 2016;1(1):1–9. https://pubmed.ncbi.nlm.nih.gov/29071308. Acessed 17 Feb 2021. PMC5653323 29071308

[24] Hatcher E, Gospodnetic O, McCandless M. Lucene in Action. 2nd ed. Manning; 2010. http://amazon.de/o/ASIN/1933988177/. Acessed 17 Feb 2021.

[25] Natukunda A, Zirimenya L, Nassuuna J, Nkurunungi G, Cose S, Elliott AM, Webb EL. The effects of helminth infection on vaccine responses in humans and animal models: a systematic review and meta-analysis. In Parasite Immunology (Vol. 44, Issue 9). John Wiley and Sons Inc. 10.1111/pim.12939. 10.1111/pim.12939 PMC9542036 35712983

[26] Appenzeller-Herzog C, Mathes T, Heeres MLS, Weiss KH, Houwen RHJ, Ewald H. Comparative effectiveness of common therapies for Wilson disease: A systematic review and meta-analysis of controlled studies. Liver International. 2019;39(11):2136–52. 10.1111/liv.14179. https://onlinelibrary.wiley.com/doi/abs/10.1111/liv.14179. 10.1111/liv.14179 31206982

[27] Appenzeller-Herzog C (2020). Data from Comparative effectiveness of common therapies for Wilson disease: A systematic review and meta-analysis of controlled studies..

[28] Feinerer I, Hornik K, Meyer D. Text Mining Infrastructure in R. J Stat Softw. 2008;25(5). 10.18637/jss.v025.i05.

[29] Gelman A, Carlin JB, Stern HS, Dunson DB, Vehtari A, Rubin DB. Bayesian Data Analysis. Chapman and Hall/CRC; 2013. 10.1201/b16018.

[30] Grün B, Hornik K. topicmodels: An R Package for Fitting Topic Models. J Stat Softw. 2011;40(13). 10.18637/jss.v040.i13.

[31] Nikita M. ldatuning: Tuning of the Latent Dirichlet Allocation Models Parameters. 2020. R package version 1.0.2. https://CRAN.R-project.org/package=ldatuning. Acessed 17 Feb 2021.

[32] Griffiths TL, Steyvers M. Finding scientific topics. Proc Natl Acad Sci. 2004;101(suppl 1):5228–35. 10.1073/pnas.0307752101. https://www.pnas.org/content/101/suppl_1/5228. 10.1073/pnas.0307752101 PMC387300 14872004

[33] Cao J, Xia T, Li J, Zhang Y, Tang S. A density-based method for adaptive LDA model selection. Neurocomputing. 2009;72(7):1775–1781. Advances in Machine Learning and Computational Intelligence. 10.1016/j.neucom.2008.06.011.

[34] Arun R, Suresh V, Veni Madhavan CE, Narasimha Murthy MN, Zaki MJ, Yu JX, Ravindran B, Pudi V (2010). On Finding the Natural Number of Topics with Latent Dirichlet Allocation: Some Observations. Advances in Knowledge Discovery and Data Mining.

