# Supplementary figures and images for: Unsupervised title and abstract screening for systematic review: a retrospective case-study using topic modelling methodology
Source: Syst Rev. 2023 Jan 3;12:1. doi: 10.1186/s13643-022-02163-4 (PMC9811792; doi:10.1186/s13643-022-02163-4)

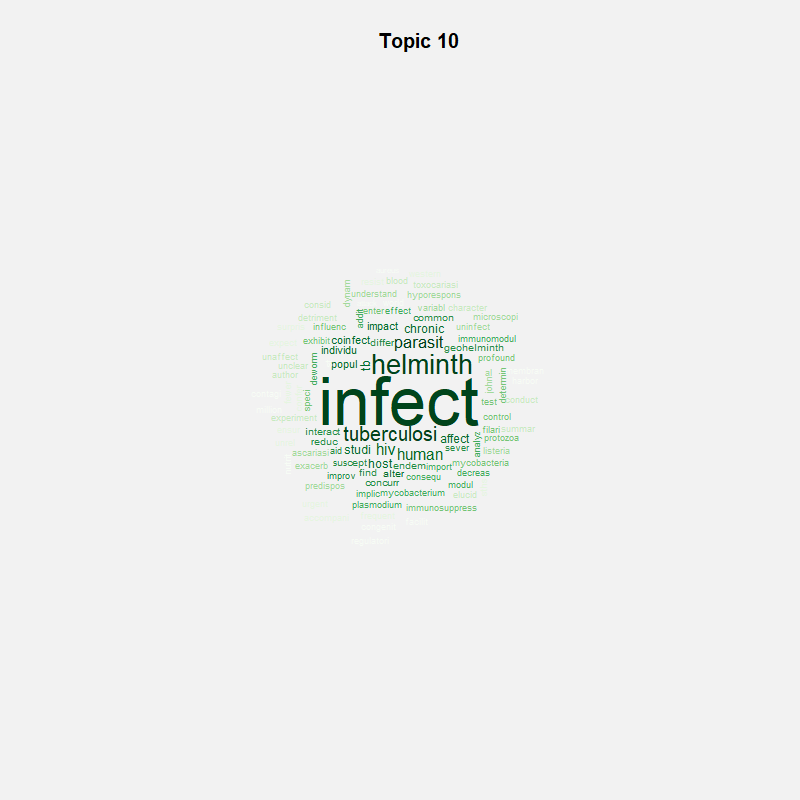

Supplement: Supplementary file 1 — Additional file 1. Zip file containing visualization of the words comprising derived LDA topics for the helminths dataset. [file 13643_2022_2163_MOESM1_ESM.zip › Topic10R2.png]

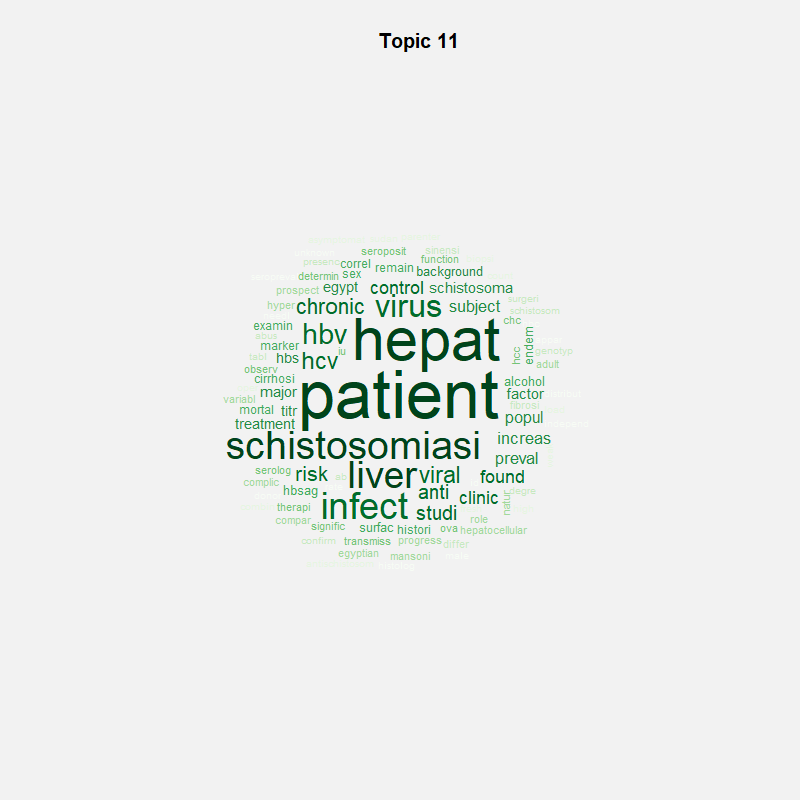

Supplement: Supplementary file 1 — Additional file 1. Zip file containing visualization of the words comprising derived LDA topics for the helminths dataset. [file 13643_2022_2163_MOESM1_ESM.zip › Topic11R2.png]

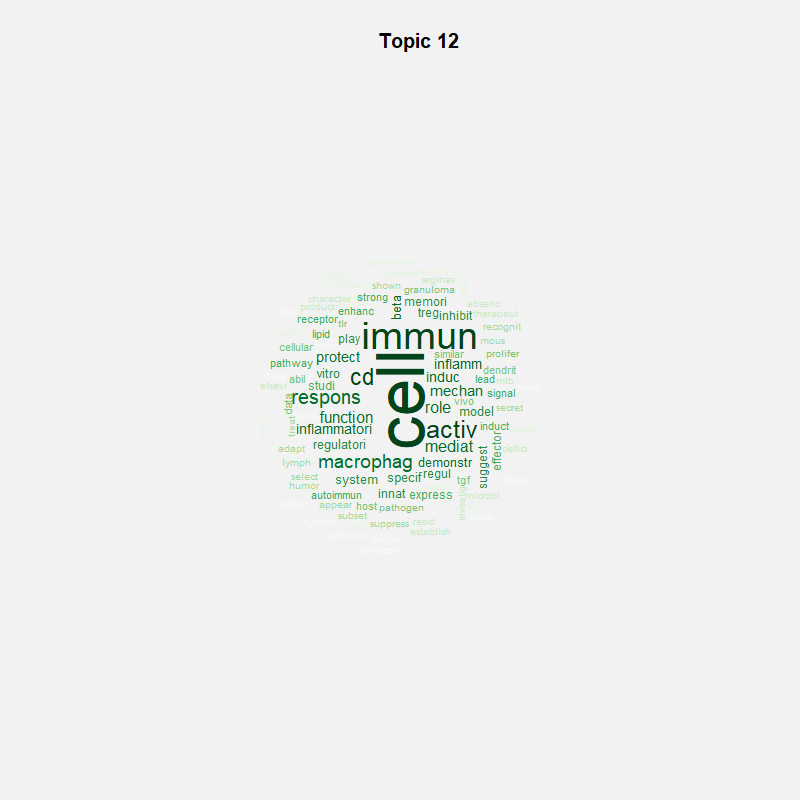

Supplement: Supplementary file 1 — Additional file 1. Zip file containing visualization of the words comprising derived LDA topics for the helminths dataset. [file 13643_2022_2163_MOESM1_ESM.zip › Topic12R2.png]

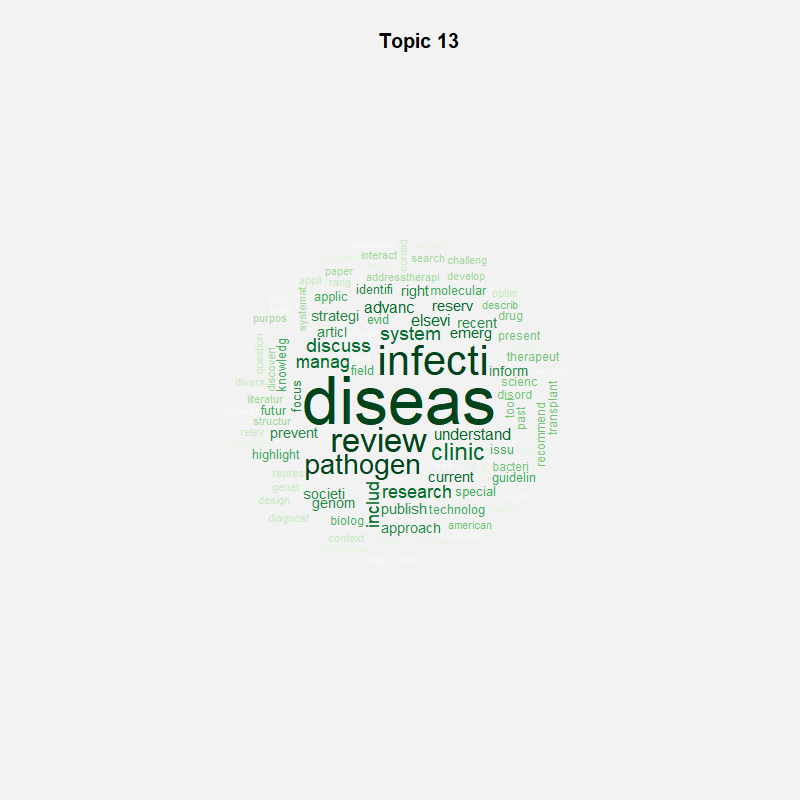

Supplement: Supplementary file 1 — Additional file 1. Zip file containing visualization of the words comprising derived LDA topics for the helminths dataset. [file 13643_2022_2163_MOESM1_ESM.zip › Topic13R2.png]

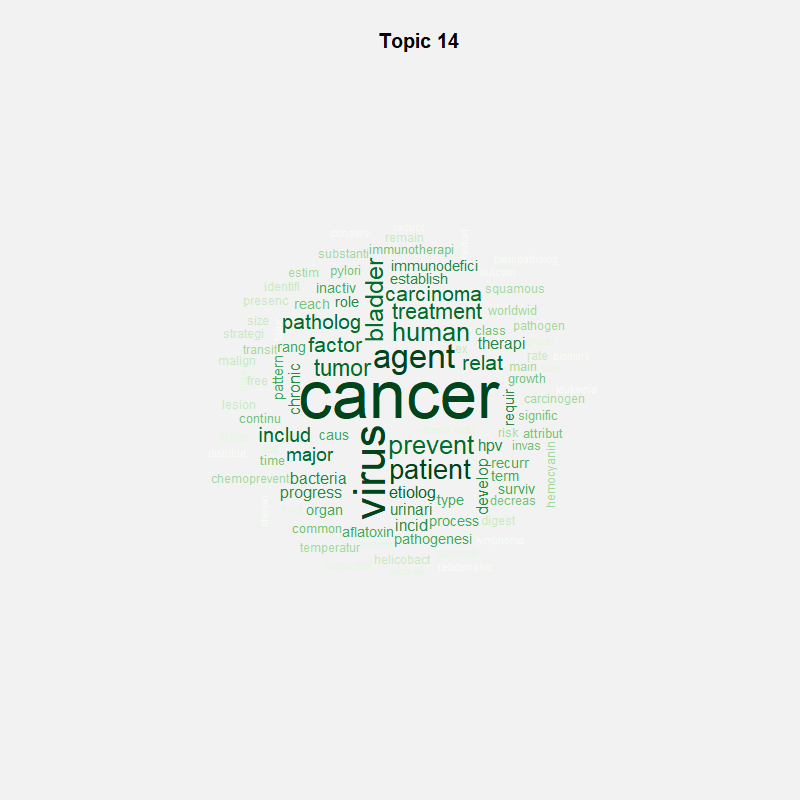

Supplement: Supplementary file 1 — Additional file 1. Zip file containing visualization of the words comprising derived LDA topics for the helminths dataset. [file 13643_2022_2163_MOESM1_ESM.zip › Topic14R2.png]

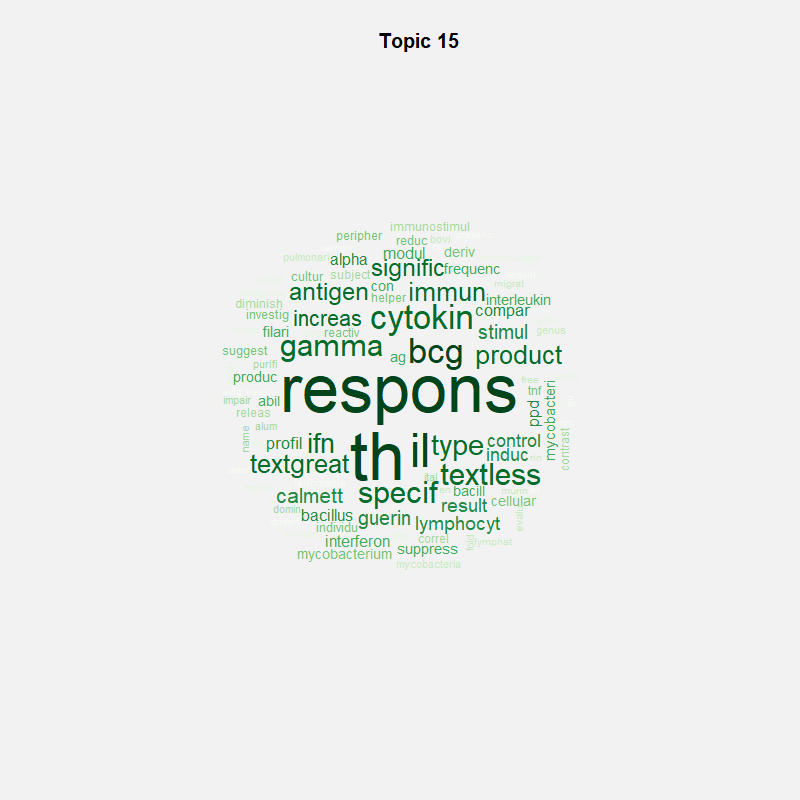

Supplement: Supplementary file 1 — Additional file 1. Zip file containing visualization of the words comprising derived LDA topics for the helminths dataset. [file 13643_2022_2163_MOESM1_ESM.zip › Topic15R2.png]

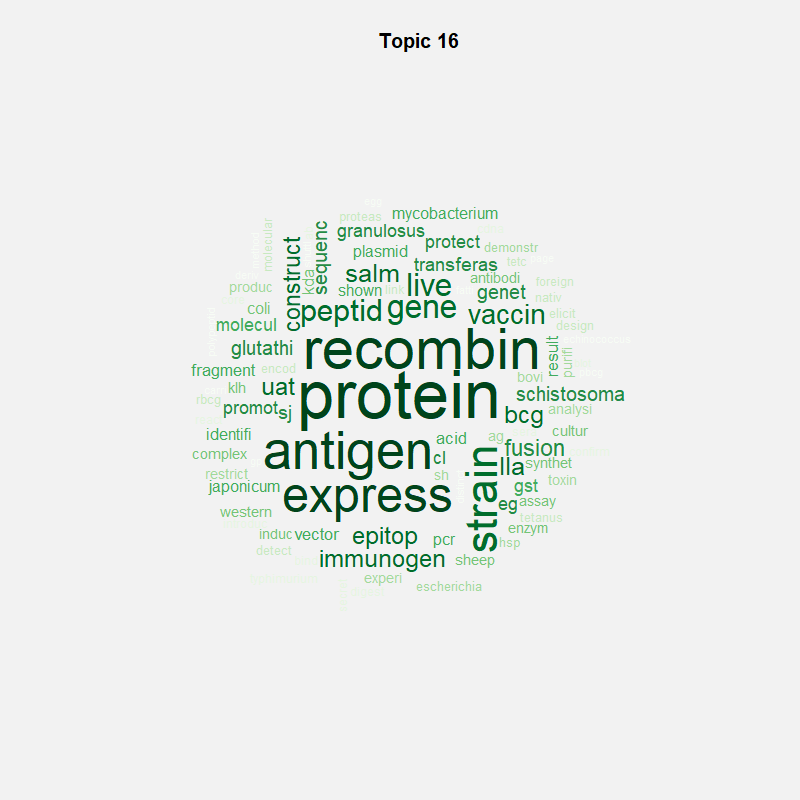

Supplement: Supplementary file 1 — Additional file 1. Zip file containing visualization of the words comprising derived LDA topics for the helminths dataset. [file 13643_2022_2163_MOESM1_ESM.zip › Topic16R2.png]

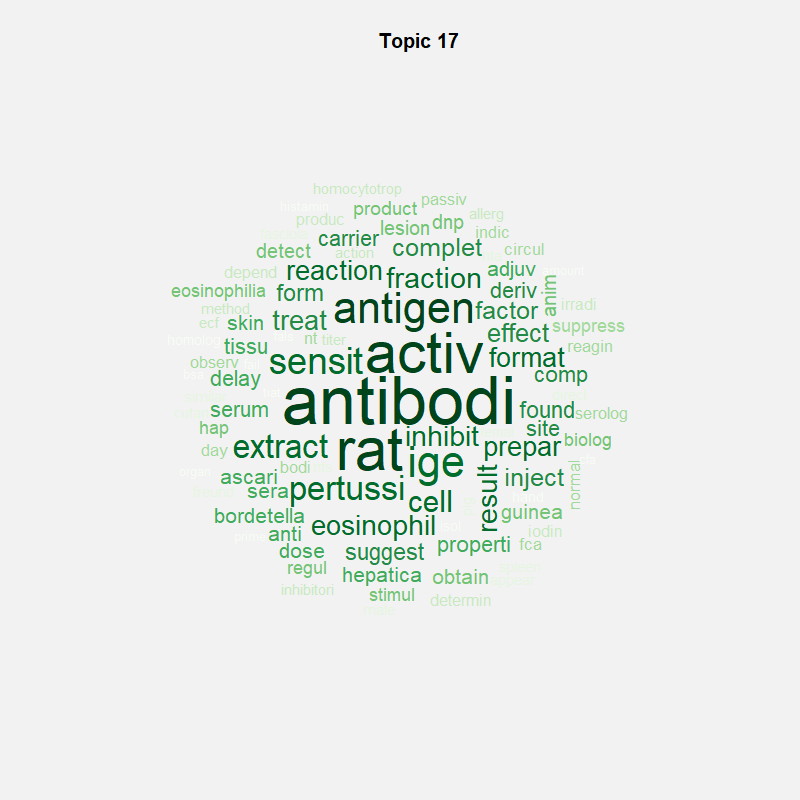

Supplement: Supplementary file 1 — Additional file 1. Zip file containing visualization of the words comprising derived LDA topics for the helminths dataset. [file 13643_2022_2163_MOESM1_ESM.zip › Topic17R2.png]

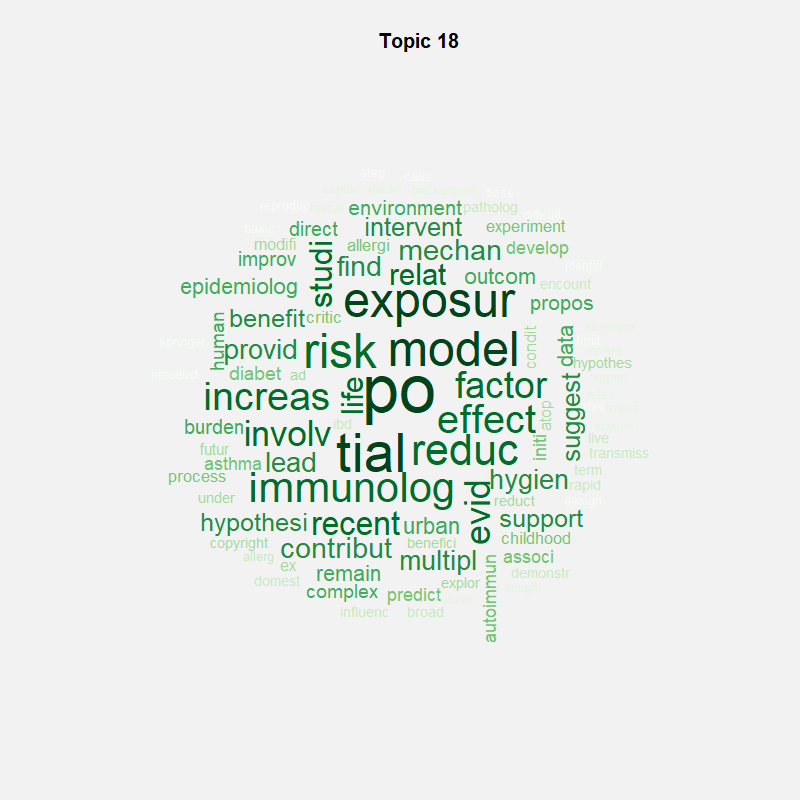

Supplement: Supplementary file 1 — Additional file 1. Zip file containing visualization of the words comprising derived LDA topics for the helminths dataset. [file 13643_2022_2163_MOESM1_ESM.zip › Topic18R2.png]

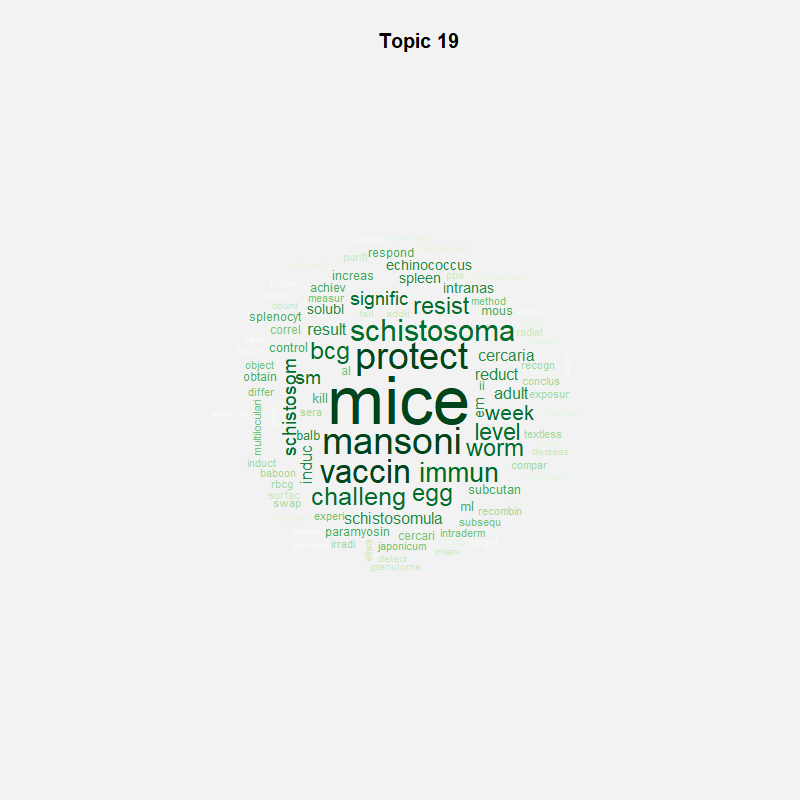

Supplement: Supplementary file 1 — Additional file 1. Zip file containing visualization of the words comprising derived LDA topics for the helminths dataset. [file 13643_2022_2163_MOESM1_ESM.zip › Topic19R2.png]

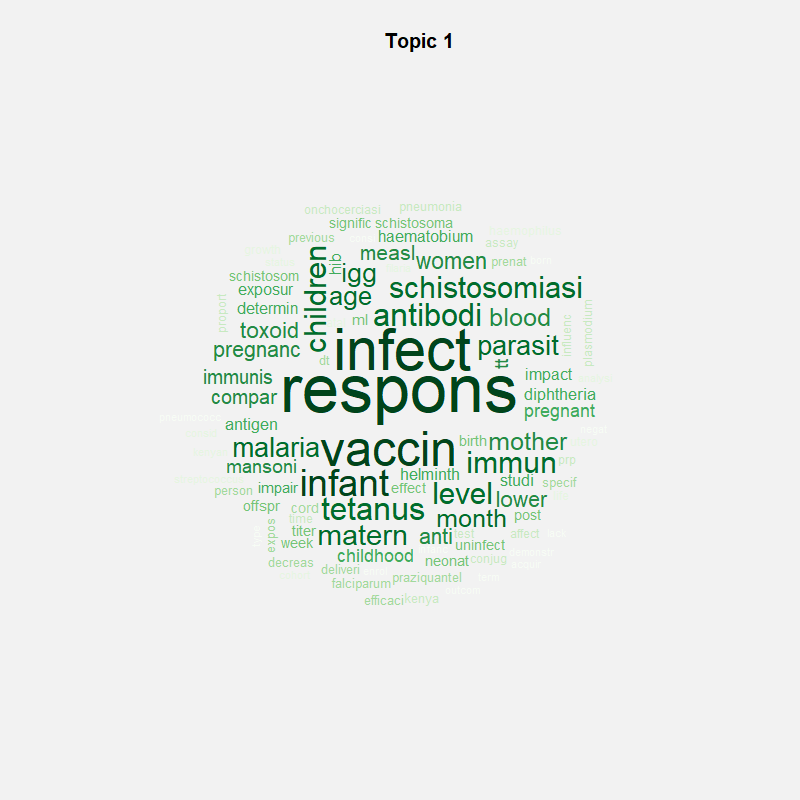

Supplement: Supplementary file 1 — Additional file 1. Zip file containing visualization of the words comprising derived LDA topics for the helminths dataset. [file 13643_2022_2163_MOESM1_ESM.zip › Topic1R2.png]

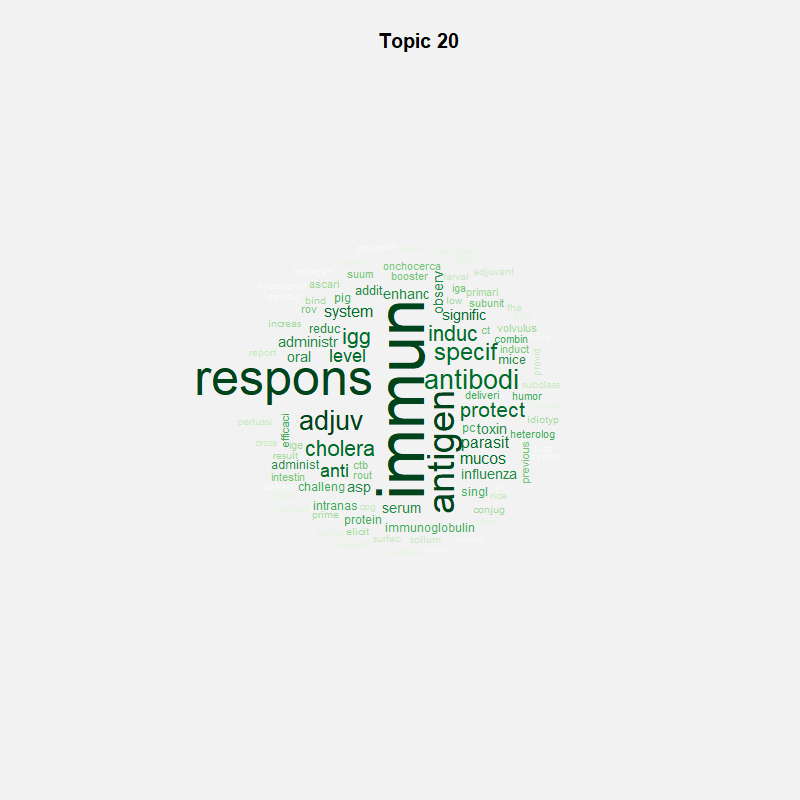

Supplement: Supplementary file 1 — Additional file 1. Zip file containing visualization of the words comprising derived LDA topics for the helminths dataset. [file 13643_2022_2163_MOESM1_ESM.zip › Topic20R2.png]

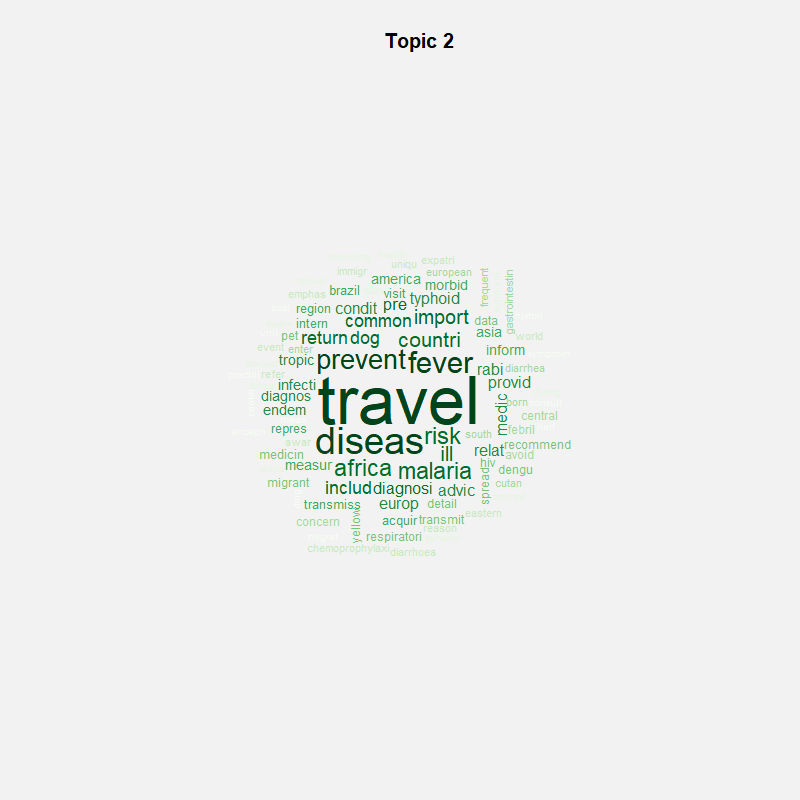

Supplement: Supplementary file 1 — Additional file 1. Zip file containing visualization of the words comprising derived LDA topics for the helminths dataset. [file 13643_2022_2163_MOESM1_ESM.zip › Topic2R2.png]

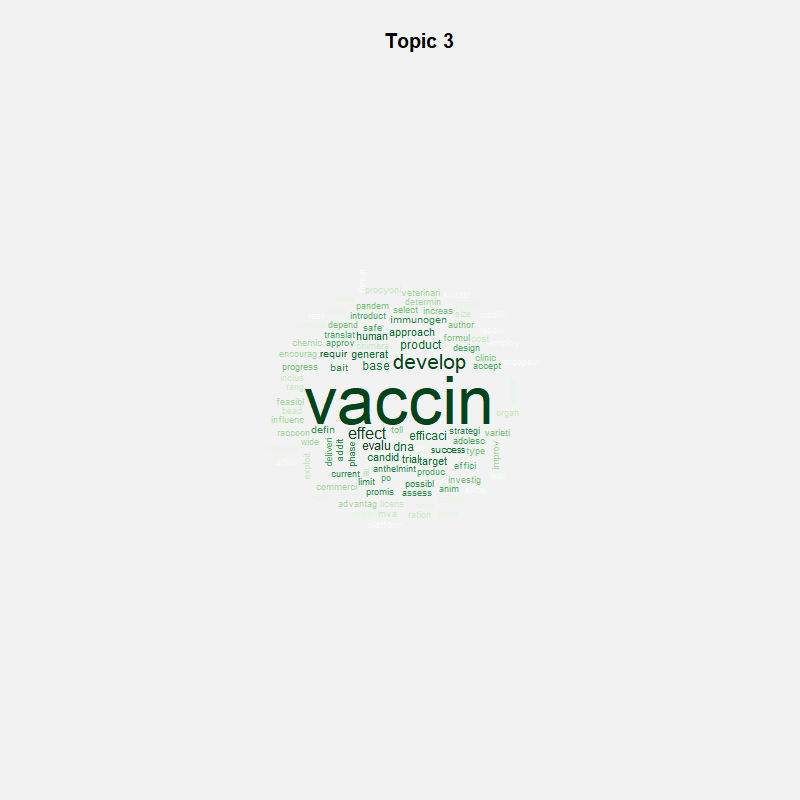

Supplement: Supplementary file 1 — Additional file 1. Zip file containing visualization of the words comprising derived LDA topics for the helminths dataset. [file 13643_2022_2163_MOESM1_ESM.zip › Topic3R2.png]

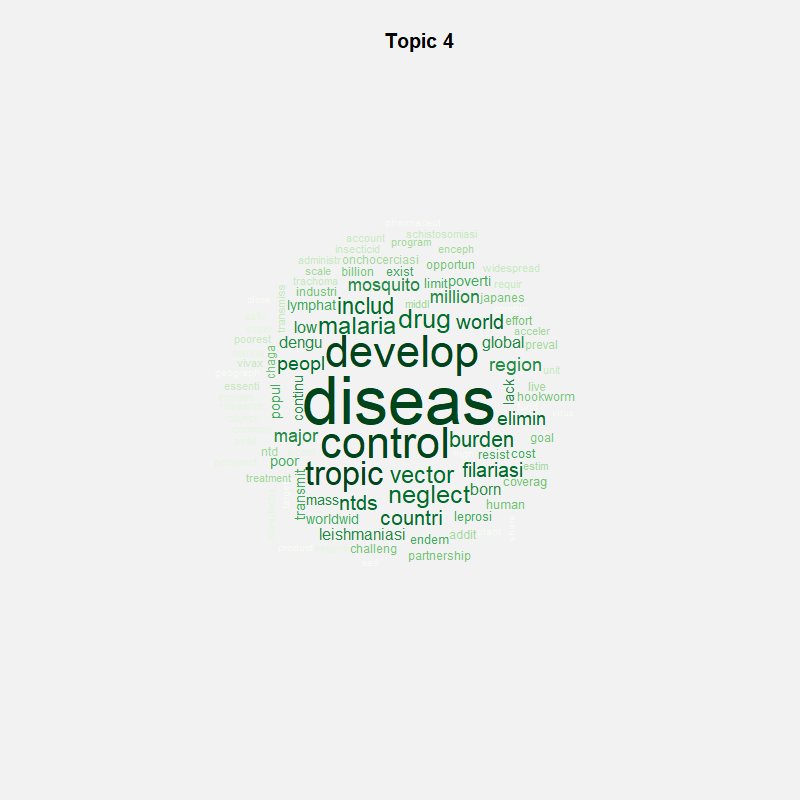

Supplement: Supplementary file 1 — Additional file 1. Zip file containing visualization of the words comprising derived LDA topics for the helminths dataset. [file 13643_2022_2163_MOESM1_ESM.zip › Topic4R2.png]

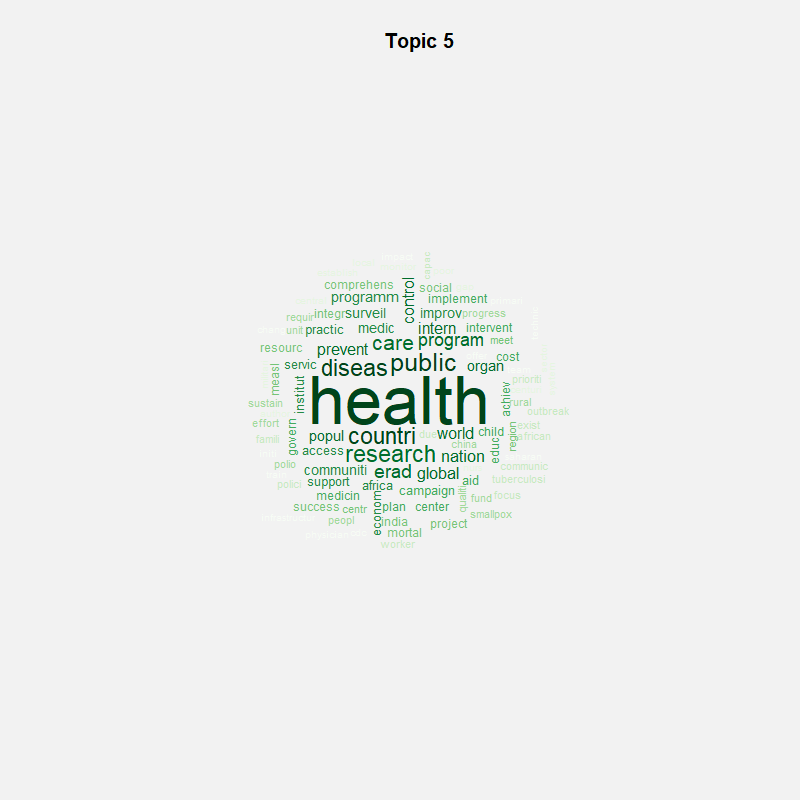

Supplement: Supplementary file 1 — Additional file 1. Zip file containing visualization of the words comprising derived LDA topics for the helminths dataset. [file 13643_2022_2163_MOESM1_ESM.zip › Topic5R2.png]

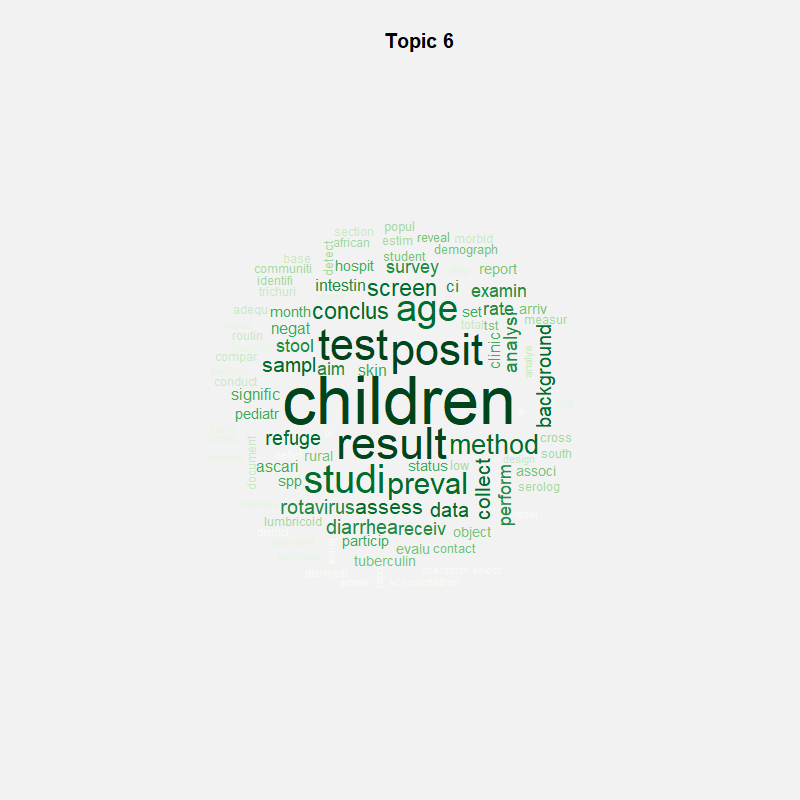

Supplement: Supplementary file 1 — Additional file 1. Zip file containing visualization of the words comprising derived LDA topics for the helminths dataset. [file 13643_2022_2163_MOESM1_ESM.zip › Topic6R2.png]

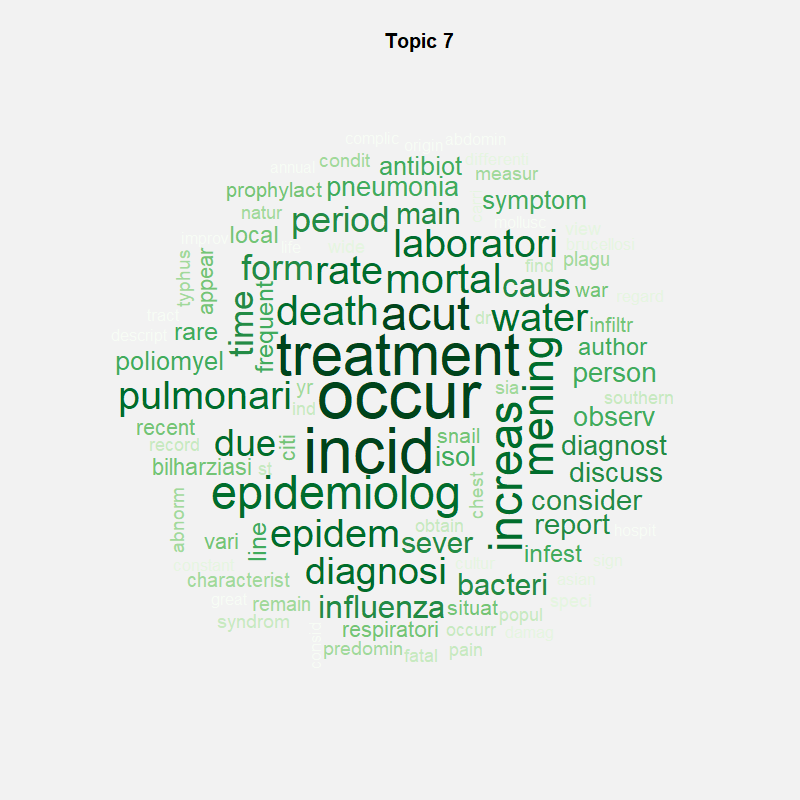

Supplement: Supplementary file 1 — Additional file 1. Zip file containing visualization of the words comprising derived LDA topics for the helminths dataset. [file 13643_2022_2163_MOESM1_ESM.zip › Topic7R2.png]

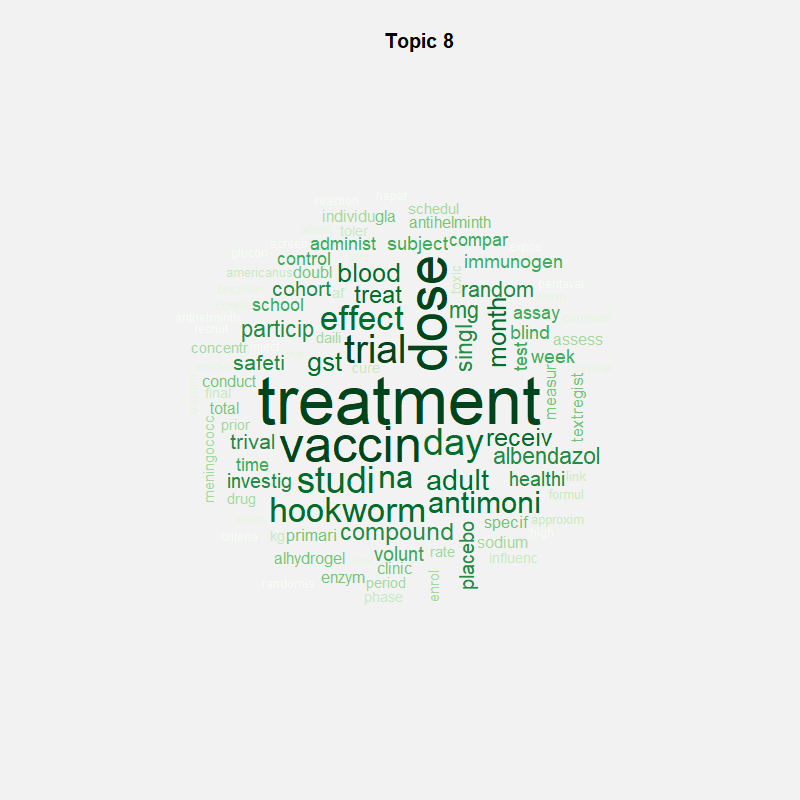

Supplement: Supplementary file 1 — Additional file 1. Zip file containing visualization of the words comprising derived LDA topics for the helminths dataset. [file 13643_2022_2163_MOESM1_ESM.zip › Topic8R2.png]

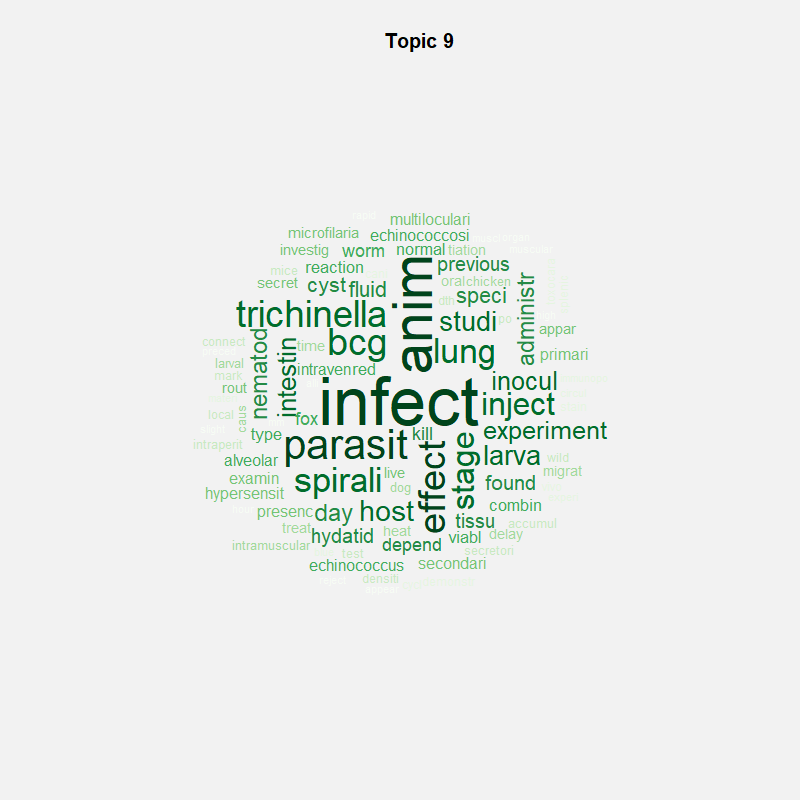

Supplement: Supplementary file 1 — Additional file 1. Zip file containing visualization of the words comprising derived LDA topics for the helminths dataset. [file 13643_2022_2163_MOESM1_ESM.zip › Topic9R2.png]
