# Supplementary material for: Unsupervised title and abstract screening for systematic review: a retrospective case-study using topic modelling methodology
Source: Syst Rev. 2023 Jan 3;12:1. doi: 10.1186/s13643-022-02163-4 (PMC9811792; doi:10.1186/s13643-022-02163-4)
Supplement: Supplementary file 1 — Additional file 1. Zip file containing visualization of the words comprising derived LDA topics for the helminths dataset. [file 13643_2022_2163_MOESM1_ESM.zip › Cover LetterR2.docx]

Systematic reviews are a commonly used tool to synthesize available published knowledge on a topic of interest. The classical approach to screening of retrieved documents title and abstract involves a time-consuming manual step and results in a significant proportion of the initially retrieved documents being excluded from further review. In this manuscript, we propose an unsupervised automated approach to screening documents for systematic review. The fundamental advantages of our proposals are 1) Unlike in supervised learning approaches, no pre-labelled training data is required to classify newly retrieved documents, hence easily adaptable to new research domains and 2) The inputs required to implement the proposed methodology are standard information that’s classically available when conducting systematic reviews (a collection of retrieved documents title and abstract as well as systematic review search and inclusion criteria). The methodology is also easily implemented in opensource software, and we provide an example workflow to help readers tailor it to their analysis pipeline. Publishing this manuscript in *Systematic Reviews* will reach a broad target audience that desperately need automated tools to more efficiently screen for documents.

The authors have reviewed the journal policies and declare that to the best of their knowledge, the manuscript does not contravene any of the polices neither do the authors have any competing interests to declare. This is a new manuscript which has not been presented or published in any form elsewhere. The authors meet authorship criteria and they have prepared, reviewed and approved the manuscript for submission to this journal.
